# Optimal signal detection with neuronal diversity: balancing the gullible and the prudent neurons

**DOI:** 10.1186/1471-2202-16-S1-P208

**Published:** 2015-12-18

**Authors:** Leonardo L Gollo, Mauro Copelli, James A Roberts

**Affiliations:** 1 Systems Neuroscience Group, QIMR Berghofer, Brisbane, Queensland, Australia; 2 Departmento de Física, Universidade Federal de Pernambuco, Recife, Pernambuco, Brazil

## 

**Figure 1:**
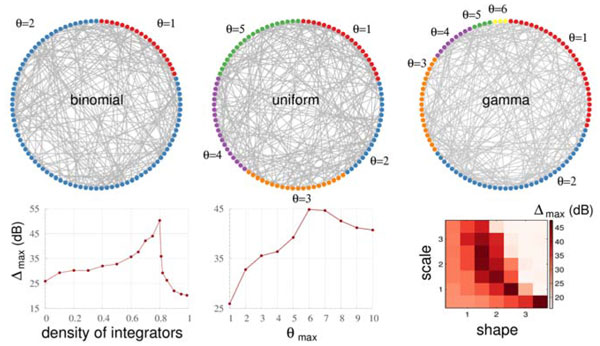
**Top: Illustrative random networks with neuronal diversity in the threshold parameter θ**. Bottom: Maximal dynamic range Δmax (as defined in ref. [6]) reached by networks with binomial (left), uniform (center), and gamma (right) distributions (bottom). Networks with binomial distribution have a proportion of integrator neurons with θ=2, whereas the remainders are non-integrator neurons (θ=1). The threshold In the uniform distribution varies from 1 to θmax. Network size is 5000 neurons.

Network connectivity have been shown to play an important role in shaping the neuronal dynamics [1-5]. A complementary remarkable feature of neuronal systems is the large degree of morphological and functional diversity. Despite some recent efforts in understanding the role of neuronal diversity embedded in a network [6-9], the benefits of cellular variability to distinguish input varying over orders of magnitude remain elusive. We utilize a simple quiescent-active-refractory-quiescent model, which is amenable to mathematical analysis [10], interacting in a (non-structured) random network with diversity in the parameter that controls the propensity of the neurons to fire in response to input from their neighbors. We consider a simple binomial distribution, a uniform distribution, and a more realistic gamma distribution. As depicted in Figure 1, we show that the capability of the network to distinguish the amount of external input can be improved by two orders of magnitude (20 dB) in the presence of diversity. We explain how diversity enhances the network capabilities, and identify the cases in which one specialized sub-population outperforms the rest of the network and the cases in which the average network outperforms any sub-population. Finally, we show the robustness of our results in a balanced cortical network of excitatory and inhibitory neurons.
